# Studying individual risk factors for self-harm in the UK Biobank: A polygenic scoring and Mendelian randomisation study

**DOI:** 10.1371/journal.pmed.1003137

**Published:** 2020-06-01

**Authors:** Kai Xiang Lim, Frühling Rijsdijk, Saskia P. Hagenaars, Adam Socrates, Shing Wan Choi, Jonathan R. I. Coleman, Kylie P. Glanville, Cathryn M. Lewis, Jean-Baptiste Pingault

**Affiliations:** 1 Social, Genetic and Developmental Psychiatry Centre, Institute of Psychiatry, Psychology & Neuroscience, King’s College London, London, United Kingdom; 2 Icahn School of Medicine at Mount Sinai, New York, New York, United States of America; 3 Division of Psychology and Language Sciences, University College London, London, United Kingdom; Columbia University, UNITED STATES

## Abstract

**Background:**

Identifying causal risk factors for self-harm is essential to inform preventive interventions. Epidemiological studies have identified risk factors associated with self-harm, but these associations can be subject to confounding. By implementing genetically informed methods to better account for confounding, this study aimed to better identify plausible causal risk factors for self-harm.

**Methods and findings:**

Using summary statistics from 24 genome-wide association studies (GWASs) comprising 16,067 to 322,154 individuals, polygenic scores (PSs) were generated to index 24 possible individual risk factors for self-harm (i.e., mental health vulnerabilities, substance use, cognitive traits, personality traits, and physical traits) among a subset of UK Biobank participants (*N* = 125,925, 56.2% female) who completed an online mental health questionnaire in the period from 13 July 2016 to 27 July 2017. In total, 5,520 (4.4%) of these participants reported having self-harmed in their lifetime. In binomial regression models, PSs indexing 6 risk factors (major depressive disorder [MDD], attention deficit/hyperactivity disorder [ADHD], bipolar disorder, schizophrenia, alcohol dependence disorder, and lifetime cannabis use) predicted self-harm, with effect sizes ranging from odds ratio (OR) = 1.05 (95% CI 1.02 to 1.07, *q* = 0.008) for lifetime cannabis use to OR = 1.20 (95% CI 1.16 to 1.23, *q* = 1.33 × 10^−35^) for MDD. No systematic differences emerged between suicidal and non-suicidal self-harm. To further probe causal relationships, two-sample Mendelian randomisation (MR) analyses were conducted, with MDD, ADHD, and schizophrenia emerging as the most plausible causal risk factors for self-harm. The genetic liabilities for MDD and schizophrenia were associated with self-harm independently of diagnosis and medication. Main limitations include the lack of representativeness of the UK Biobank sample, that self-harm was self-reported, and the limited power of some of the included GWASs, potentially leading to possible type II error.

**Conclusions:**

In addition to confirming the role of MDD, we demonstrate that ADHD and schizophrenia likely play a role in the aetiology of self-harm using multivariate genetic designs for causal inference. Among the many individual risk factors we simultaneously considered, our findings suggest that systematic detection and treatment of core psychiatric symptoms, including psychotic and impulsivity symptoms, may be beneficial among people at risk for self-harm.

## Abstract 2

Kai Xiang Lim and co-workers use genetic instruments to investigate risk factors associated with self-harm.

## Author summary

**Why was this study done?:**

- Self-harm is an act of self-injury or self-poisoning carried out by an individual and can be subdivided into suicidal and non-suicidal self-harm.
- Self-harm has been found to be associated with a range of risk factors, but these associations can be subject to confounding.
- Using genetic information to help strengthen causal inference can help to identify plausibly causal risk factors for self-harm.

**What did the researchers do and find?:**

- We used data from UK Biobank participants, who provided their genetic information and answered questions about their engagement in self-harming.
- We investigated 24 potential individual risk factors for self-harm.
- Out of these 24 risk factors, major depressive disorder, attention-deficit hyperactivity disorder and schizophrenia appeared to be the most plausible causal risk factors for self-harm.
- No difference emerged between risk factors for non-suicidal and suicidal self-harm.

**What do these findings mean?:**

- Using a different design, with different sets of assumptions, than epidemiological studies that are not genetically informed, we showed that psychiatric symptoms related to major depressive disorder, attention deficit/hyperactivity disorder, and schizophrenia plausibly play a role as individual risk factors leading to an increased risk of self-harm.
- Detecting and treating psychiatric symptoms, such as impulsivity and psychotic symptoms, may be helpful for people at risk for self-harm.

## Introduction

Self-harm refers to any act of self-injury or self-poisoning carried out by an individual, regardless of intention or motivation [1]. It can be further categorised into suicidal self-harm (SSH) and non-suicidal self-harm (NSSH). To illustrate, NSSH is an intentional act of harming oneself without suicidal intention, such as self-cutting, self-burning, and self-poisoning [2]. SSH, on the other hand, is associated with the intention to die, such as overdosing, severe cutting, and hanging/strangulation, and is often more lethal than NSSH [3]. According to a meta-analysis, the cross-national prevalence rate for NSSH peaks during adolescence (17.3%) and decreases among adults (5.5%) [4]. For SSH, the cross-national prevalence rate is also highest among adolescents (9.7%) [5] and drops among adults (2.7%) [6]. Recently, the fifth edition of the Diagnostic and Statistical Manual of Mental Disorders (DSM-5) divided self-harm along the dimension of suicidal intention as 2 separate conditions for further study [7]. The distinction between SSH and NSSH may facilitate investigations of the aetiology and heterogeneity of self-harm.

Multiple individual vulnerabilities and traits can potentially lead to self-harm, such as psychiatric illnesses [8], substance use [9–11], cognitive abilities [12], personality traits [13], and physical traits [14]. The stress–diathesis model of suicidal behaviour proposes that the risk for suicidal behaviours is determined not just by stressors such as major psychiatric disorders, but also by a diathesis, rooted in susceptibilities such as cognitive abilities and impulsivity [15–18]. Although associations between these risk factors and self-harm have been shown in numerous observational studies, causality is difficult to infer reliably. Genetically informed designs can help strengthen causal inference [19]. A polygenic score (PS) is a single individual-level score computed for a given trait, weighted using summary statistics from an independent genome-wide association study (GWAS) for that particular trait. A PS for an individual risk factor (e.g., schizophrenia) can be regarded as a genetic proxy for this risk factor [20]. To illustrate, if schizophrenia is causally related to self-harm, a PS for schizophrenia should also be associated with self-harm. A significant association between the PS for schizophrenia and self-harm can be regarded as an initial indication of a possible causal relationship between the 2 traits. The PS approach can be construed as a first step in a series of genetically informed methods to investigate the aetiology of complex phenotypes, with follow-up steps including Mendelian randomisation (MR), discussed below [19–21].

In previous studies, a PS for major depressive disorder (MDD) predicted SSH in 2 clinical samples [22,23] and 1 non-clinical sample [24]. However, this finding was not replicated in a family-based sample after applying Bonferroni correction (although, at the nominal *p*-value threshold, the PS for MDD generated from the first Psychiatric Genomics Consortium GWAS for MDD predicted SSH among MDD cases in this sample) [25]. A PS for depressive symptoms predicted SSH but not NSSH in a twin sample [26]. A PS for schizophrenia predicted SSH among offspring of suicide attempters [25] and in a population sample [27], but not in another clinical sample [28]. A PS for bipolar disorder predicted SSH in 1 clinical sample [29] but did not predict SSH nor NSSH among offspring of suicide attempters [25] or relatives of bipolar disorder patients [30]. A recently published study showed that PSs for cannabis use and MDD predicted self-harm in the UK Biobank sample [31].

The aforementioned PS studies with mixed results were limited in several ways. First, these studies mostly focused on PSs for psychiatric disorders or symptoms, and did not include potential individual risk factors from other domains, such as substance use [9–11], cognitive abilities [12], personality traits [13], and physical traits [14]. Second, with 2 exceptions [26,30], none of the studies investigated SSH and NSSH simultaneously. Third, these studies have a mixture of clinical and non-clinical samples with varying sample sizes ranging from 224 individuals [28] to 63,054 individuals [31], making any comparison difficult. Furthermore, none of these studies have implemented multivariate analyses with multiple PSs to better estimate their unique effect.

A caveat of the PS method is its proneness to unmediated (or horizontal) pleiotropy, arising from the inclusion of thousands of genetic variants [20]. Unmediated pleiotropy exists when a genetic variant associated with an exposure causes the outcome through an alternative pathway, instead of via the exposure. Unmediated pleiotropy can generate associations between PSs and outcomes in the absence of a causal relationship between the risk factors indexed by the PS, and the outcome. MR can more stringently address unmediated pleiotropy and further strengthen causal inference. In MR, individual genetic variants associated with an exposure of interest are used as instrumental variables to infer causality between exposure and outcome. A number of complementary analyses, further detailed in the Methods section, can be implemented to account for pleiotropy [19]. To date, to our knowledge, there is no published MR study that focuses on any risk factor for self-harm.

The current study addressed the aforementioned limitations by systematically using 24 PSs as proxies for risk factors from different domains to predict both NSSH and SSH, using a population-based sample of 125,925 individuals from the UK Biobank. Selected PSs reflect in part the 2 dimensions of the stress–diathesis model of suicidal behaviour [15–18]. We also conducted follow-up MR analyses to strengthen causal inference. This study builds on previous genetic studies using UK Biobank samples that have identified 3 genome-wide significant loci for suicidality [32] and found significant genetic correlations between SSH and several psychiatric disorders [33].

## Methods

This study did not have a prespecified analysis plan.

### Participants

The participants are a subset of the UK Biobank (http://www.ukbiobank.ac.uk). A total of 157,358 participants completed an online mental health questionnaire in a period from 13 July 2016 to 27 July 2017, including questions regarding their lifetime symptoms of mental disorders [34]. After genotyping and quality control processing (see S1 Method), the final sample size was 125,925 individuals (56.2% female), with ages ranging from 48 to 82 years (mean = 65.88, SD = 7.69).

The UK Biobank received ethical approval from the North West–Haydock Research Ethics Committee (REC reference 11/NW/0382). The current study was conducted under UK Biobank application 18177. Appropriate informed consent was obtained from the participants.

### Defining self-harm phenotypes

**Fig 1:**
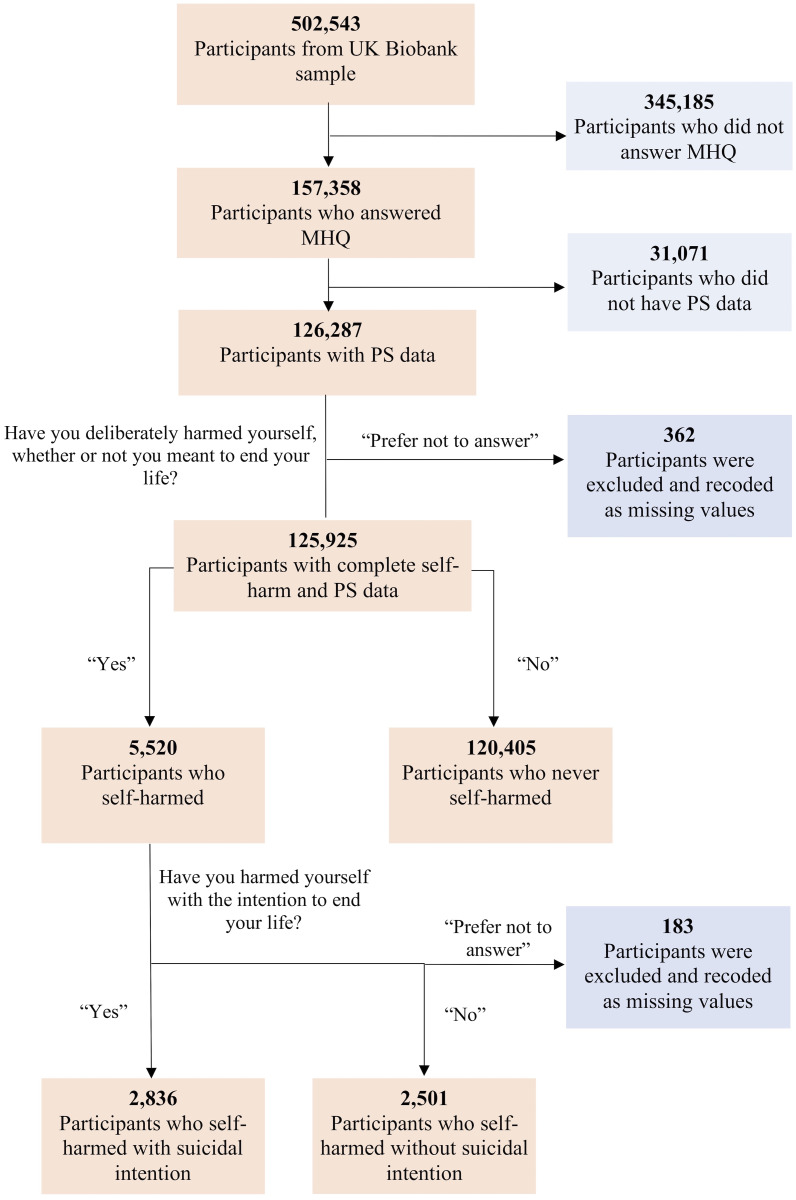
Participant flowchart. MHQ, mental health questionnaire; PS, polygenic score.

To know whether the participants have ever self-harmed, participants were asked, ‘Have you deliberately harmed yourself, whether or not you meant to end your life?’ To ascertain whether their self-harm episodes were NSSH or SSH, they were asked, ‘Have you harmed yourself with the intention to end your life?’ In both questions, responses of ‘Prefer not to answer’ (0.43%) were recoded as missing values. A flowchart depicting exclusion of participants and the number of participants who answered each question is shown in Fig 1. We used the final sample with complete data (i.e., without missing genotype or self-harm data) for subsequent analyses.

### Statistical analyses

All statistical analyses were conducted in Linux environment using R version 3.5.0 [35].

### PS analyses

PSs of UK Biobank participants were generated using PRSice-2 [36] based on their genotype data and 24 publicly available summary data from GWASs (see Table 1) selected based on the following criteria. First, we selected GWASs indexing individual vulnerabilities and traits that can potentially increase the risk of self-harm, including mental health vulnerabilities (e.g., MDD) [37], cognitive abilities (e.g., education attainment) [38], personality traits (e.g., neuroticism) [39], substance use phenotypes (e.g., cannabis use) [40], and physical traits (e.g., BMI) [14]. Second, we selected GWASs that only included participants of European ancestry and did not include UK Biobank participants (to avoid overlapping between the discovery and target samples). Finally, we excluded GWASs with effective sample sizes less than *N* = 15,000 to limit the use of underpowered PSs.

**Table 1:** Single and multiple PS prediction of self-harm.

Trait/disorder	Discovery sample size	Single PS binomial model					Multiple PS binomial model			
		OR	95% CI lower bound	95% CI upper bound	*p*-Value	*q*-Value	OR	95% CI lower bound	95% CI upper bound	*p*-Value
**Mental health vulnerabilities**										
ADHD symptoms [41]	17,666	1.031	1.003	1.059	0.030	0.074	—	—	—	—
ADHD [42]	49,017*	1.132	1.102	1.164	6.69 × 10^−19^	**8.02 × 10**^**−18**^	1.093	1.063	1.125	**4.24 × 10**^**−10**^
Alcohol dependence disorder [43]	42,803*	1.046	1.016	1.076	0.002	**0.008**	1.024	0.995	1.054	0.101
Anxiety disorders meta-analysis: Factor scores [44]	18,186	1.026	0.999	1.055	0.060	0.121	—	—	—	**—**
Anxiety disorders meta-analysis: Case–control [44]	17,310	1.022	0.995	1.050	0.116	0.199	—	—	—	—
Bipolar disorder [45]	16,544*	1.070	1.041	1.100	1.74 × 10^−6^	**1.05 × 10**^**−5**^	1.032	1.002	1.061	**0.033**
MDD [37]	124,331*	1.197	1.164	1.230	5.52 × 10^−37^	**1.33 × 10**^**−35**^	1.155	1.123	1.189	**3.99 × 10**^**−23**^
Schizophrenia [46]	75,846*	1.137	1.105	1.170	2.43 × 10^−18^	**1.94 × 10**^**−17**^	1.098	1.066	1.132	**6.99 × 10**^**−10**^
**Substance use**										
Lifetime cannabis use [40]	31,933*	1.045	1.016	1.074	0.002	**0.008**	1.036	1.008	1.066	**0.013**
Cigarettes per day [47]	38,181	0.986	0.960	1.014	0.329	0.465	—		—	—
Daily alcohol use [48]	70,460	1.001	0.969	1.034	0.959	0.991	—		—	—
**Cognitive trait**										
Education attainment [38]	106,736	1.000	0.972	1.029	0.991	0.991	—		—	—
**Personality traits**										
Conscientiousness [49]	17,375	0.991	0.964	1.019	0.524	0.599	—		—	—
Extraversion [50]	63,030	0.983	0.956	1.010	0.214	0.343	—		—	—
Neuroticism (IRT) [39]	63,661	1.032	1.004	1.060	0.025	0.074	—		—	—
Agreeableness [49]	17,375	0.989	0.962	1.016	0.414	0.523	—		—	—
Aggression [51]	18,988	1.023	0.995	1.051	0.107	0.198	—		—	—
Antisocial behaviour [52]	16,400	1.028	1.000	1.056	0.049	0.108	—		—	—
**Physical traits**										
Birth length [53]	28,459	0.992	0.965	1.020	0.592	0.646	—		—	—
Birth weight [54]	26,836	1.031	1.003	1.060	0.031	0.074	—		—	—
Adult height [55]	253,288	0.982	0.945	1.021	0.365	0.486	—		—	—
Overweight [56]	154,206*	1.011	0.984	1.039	0.440	0.527	—		—	—
Extreme BMI [56]	16,067*	1.032	1.004	1.060	0.024	0.074	—		—	—
BMI [57]	322,154	1.016	0.988	1.045	0.259	0.388	—		—	—

The *p*-values and *q*-values in bold are those that met the nominal *p* < 0.05 (in multiple PS binomial model) or corrected *q* < 0.05 (in single PS binomial model) thresholds. For the MDD GWAS, we were able to remove UK Biobank participants from the latest GWAS [37].

*GWASs with case–control samples. The effective sample sizes are calculated using the formula *N*_effective_ = 4/(1/*N*_cases_ + *N*_controls_) whenever possible.

ADHD, attention deficit/hyperactivity disorder; GWAS, genome-wide association study; IRT, item response theory; MDD, major depressive disorder; OR, odds ratio; PS, polygenic score.

Each participant had 24 PSs, which were each calculated as the sum of alleles associated with their respective phenotypes, weighted by their effect sizes, with *p*-values less than a threshold *p*_*T*_ < 0.3. Similar to previous PS studies [27,31], we used 1 single liberal threshold, which allowed us to limit multiple testing while retaining the predictive ability of included PSs [58]. Clumping was used to remove SNPs in linkage equilibrium (*r*^*2*^ < 0.1 within a 250-kb window). All PSs in the final analytical sample were standardised.

#### Single PS binomial logistic regression

For each PS, a binomial logistic regression was conducted to test whether it predicted self-harm (i.e., ‘self-harmed’ versus ‘never self-harmed’).

#### Multiple PS binomial logistic regression

All PSs significantly associated with self-harm in single PS binomial logistic regressions after correction for multiple testing were then jointly modelled in a multivariate binomial logistic regression model to assess their unique effects.

#### Single PS multinomial logistic regressions

To investigate whether each PS differentially predicted NSSH versus SSH, we fitted a series of multinomial logistic regression models. We first compared each of the NSSH and SSH groups to the never-self-harmed group (i.e., ‘never self-harmed’ as the reference group). We then directly compared the NSSH and SSH groups by testing a model with NSSH as the reference group.

#### Covariates and multiple testing

All regression models were controlled for sex, age, and population stratification (by including assessment centre, genotyping batch, and the first 6 principal components as covariates in the models). To control for multiple testing in single PS binomial and multinomial regressions, we employed the false discovery rate (FDR) method [59], which controls the expected proportion of false positives among the rejected hypotheses. We used *q* < 0.05 as the significance threshold.

### MR analyses

All MR analyses were conducted using R package TwoSampleMR [60]. Risk factors for which the PS significantly predicted self-harm were selected as exposures for follow-up MR analyses. For self-harm in the UK Biobank sample as the outcome for MR analyses, we obtained GWAS summary statistics from Neale Lab (http://www.nealelab.is/uk-biobank). Exposure SNPs that passed the *p*-value threshold of *p* < 5 × 10^−5^ were selected as instrumental variables. A liberal threshold was used to ensure that enough variants were available for all risk factors, including those with few genome-wide significant SNPs (e.g., ADHD). The strategy entails potential weak instrument bias. In two-sample MR, the resulting bias is towards the null, making estimates more conservative (see below for how this was dealt with) [61]. For more details on clumping and harmonisation, see Text A in S2 Method.

We selected 4 MR methods that have different strengths and limitations. We conducted univariable MR using (i) inverse variance weighting (IVW)—the most powerful method (but cannot account for directional pleiotropy) [62]; (ii) robust adjusted profile score (RAPS)—to account for the selection of weak instruments [63]; (iii) weighted median—more robust to directional pleiotropy than IVW, and more robust to individual genetic variants with outlying causal estimates than IVW and MR–Egger regression [64]; and (iv) MR–Egger regression—the significance of its intercept term informs on the presence of directional pleiotropy [65].

We calculated the *I*^2^_GX_ statistics for the MR instruments to assess the suitability of carrying out the MR–Egger regression method [66]. We also implemented MR Steiger filtering [67] to address the possibility of reverse causation (see Text B in S2 Method). Finally, similar to PS analyses, exposures that were significant in univariable MR were assessed for their independent effect in a multivariable MR model using the IVW method. GWAS summary statistics for SSH are also available. We thus repeated MR analyses for SSH only.

For PS analyses, we conducted further complementary analyses excluding cases with MDD and schizophrenia diagnoses to investigate the effect of genetic liability on self-harm with the influence of these diagnoses excluded. We also calculated risk ratios of self-harm for medicated and non-medicated cases compared to those with median PS in the general population (see S1 Appendix).

This study is reported as per the Strengthening the Reporting of Observational Studies in Epidemiology (STROBE) guideline (see S1 Checklist).

## Results

### Descriptive statistics

Fig 1 shows the number of participants who never self-harmed, self-harmed, engaged in SSH, and engaged in NSSH. S1 Table shows the descriptive statistics of each group.

### PS analyses

#### Single PS binomial logistic regression

**Fig 2:**
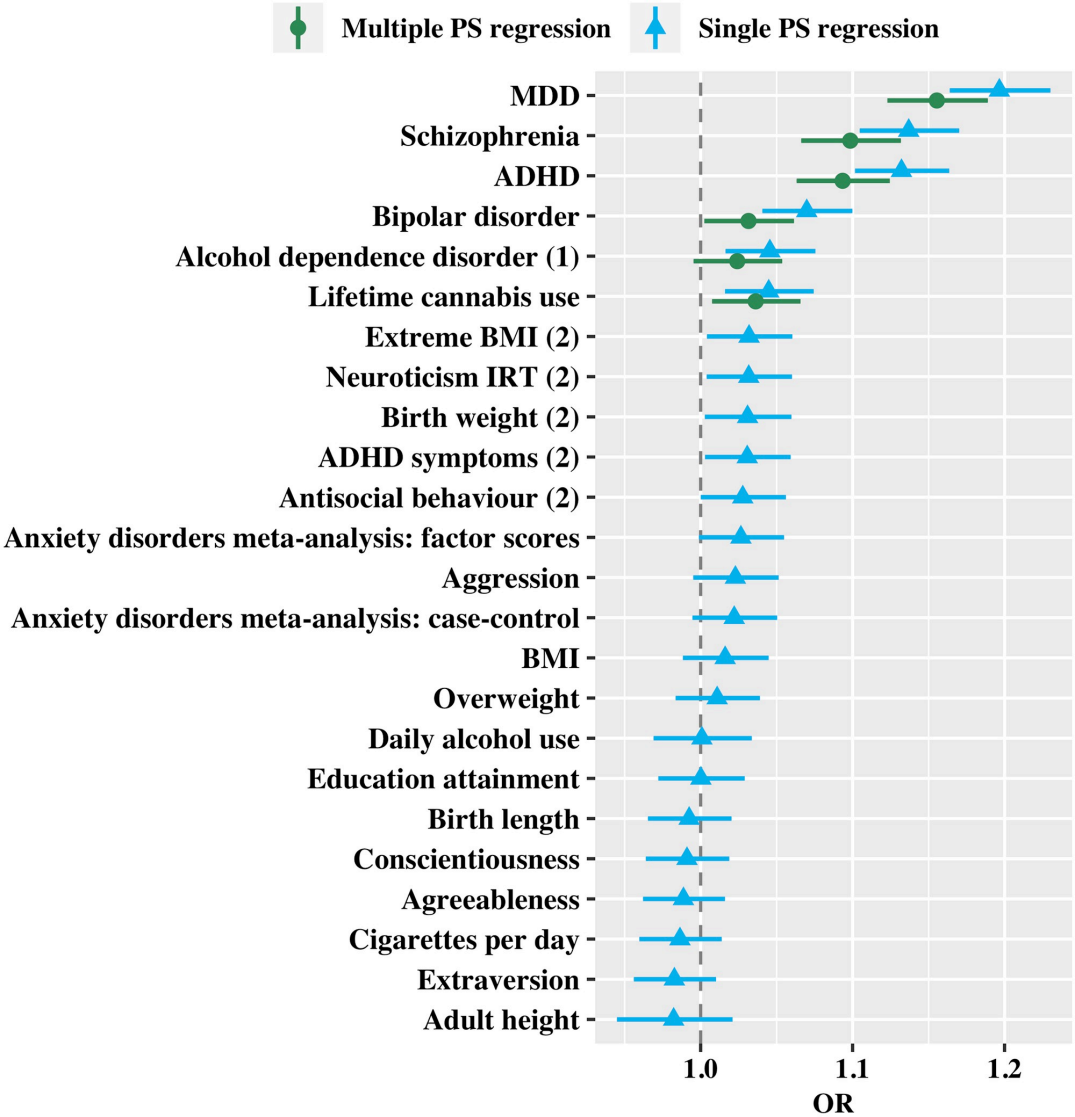
Single and multiple PS analysis of self-harm. Estimates from single PS regressions and multiple PS regression for PSs of 24 risk factors in predicting self-harm, ordered according to decreasing effect sizes. ‘(1)’ indicates not significant in multiple PS regression. ‘(2)’ indicates not significant after FDR correction. ADHD, attention deficit/hyperactivity disorder; FDR, false discovery rate; IRT, item response theory; MDD, major depressive disorder; OR, odds ratio; PS, polygenic score.

Table 1 and Fig 2 show results from 24 single PS binomial logistic regression tests, using each PS as predictor. Out of the 24 PSs, 10 PSs were significant predictors of self-harm at the nominal level (*p* < 0.05). After FDR correction, 6 PSs had *q*-value < 0.05. In order of decreasing effect sizes, they are PSs for MDD, schizophrenia, ADHD, bipolar disorder, alcohol dependence disorder (ALC), and lifetime cannabis use, with effect sizes ranging from OR = 1.197 (95% CI 1.164 to 1.230) for MDD to OR = 1.045 (95% CI 1.016 to 1.074) for lifetime cannabis use. S1 Fig shows the pseudo *R*^2^ plots of these 6 PSs in accounting for the variance in self-harm.

#### Multiple PS binomial logistic regression

In the multiple PS model, all PSs except the PS for ALC had an independent effect on self-harm, as shown in Table 1 and Fig 2. Because this model controls for the effects of other PSs, the effect sizes of these PSs are slightly lower than those in the single PS binomial logistic regressions, ranging from OR = 1.032 (95% CI 1.002 to 1.061) for bipolar disorder to OR = 1.155 (95% CI 1.123 to 1.189) for MDD. These PSs were only weakly correlated, suggesting that multicollinearity was not an issue (see S2 Table).

**Fig 3:**
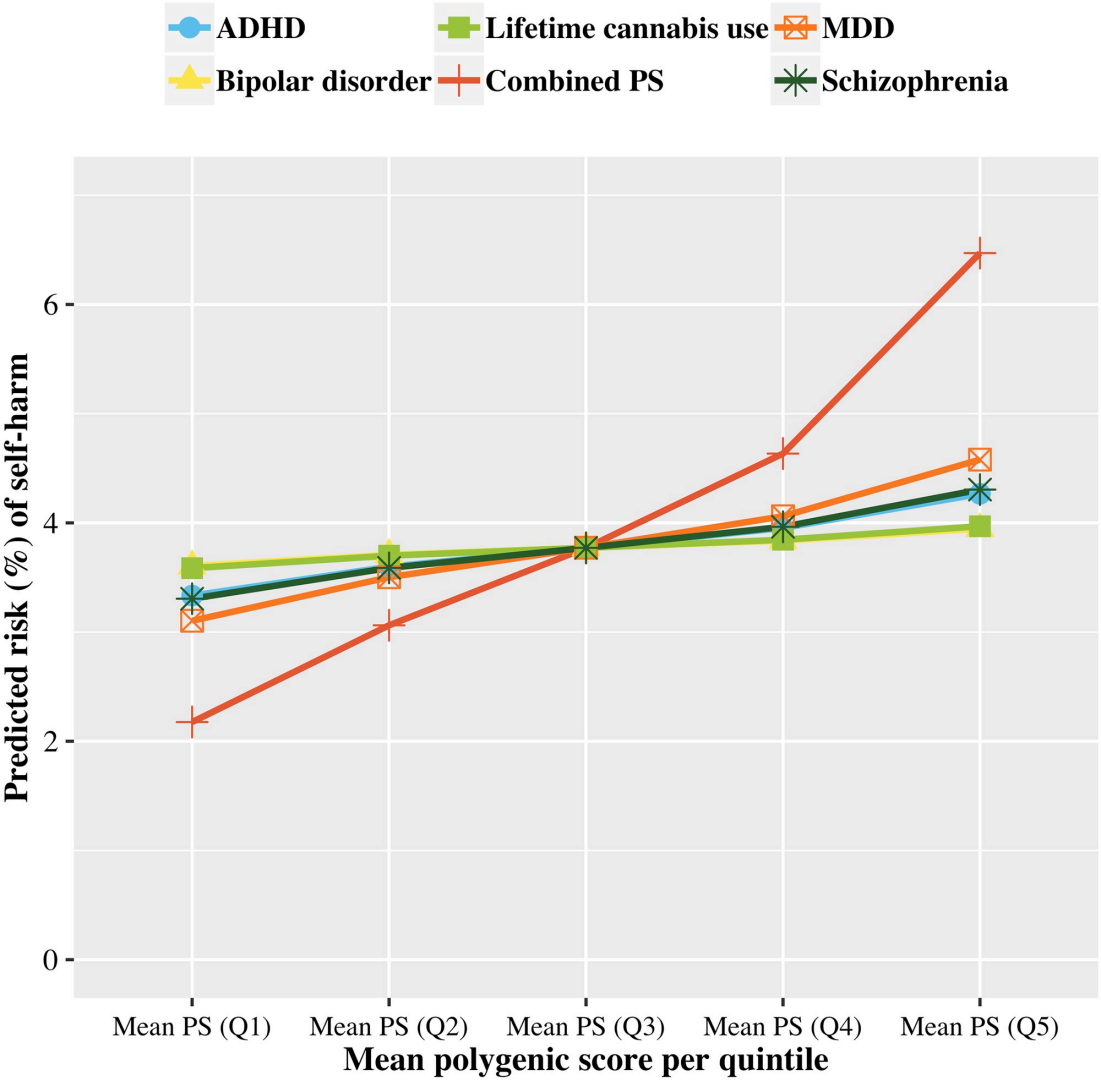
Predicted risk of self-harm for each of the 5 significantly predictive PSs and their combined predicted risk. Plot of the independently predicted risk of self-harm for each of the 5 significantly predictive PSs (for ADHD, bipolar disorder, lifetime cannabis use, MDD, and schizophrenia) and their combined predicted risk obtained from multivariate logistic regression. For independent prediction using a single PS, the mean of that particular PS in each quintile was used as predictor while the values of other PSs were set to 0 (i.e., the centred mean). For combined prediction, for each quintile, 5 mean values of the PS (mean PS for ADHD, bipolar disorder, lifetime cannabis use, MDD, and schizophrenia) were entered simultaneously as predictors. ADHD, attention deficit/hyperactivity disorder; MDD, major depressive disorder; PS, polygenic score.

Fig 3 shows the independent effects of the 5 significantly predictive PSs (MDD, schizophrenia, ADHD, bipolar disorder, and lifetime cannabis use) corresponding to the mean PS in each quintile, and their combined effect on the predicted risk of self-harm. To illustrate, for the independent effect of MDD, the predicted risk of self-harm for those whose PS for MDD was in the lowest 20% of the sample was 3.10% (95% CI 2.95% to 3.26%), as compared to 4.58% (95% CI 4.37% to 4.79%) for those whose PS for MDD was in the highest 20%. For combined effect prediction, among participants whose 5 PSs were all in the lowest 20% of the sample, their combined predicted risk of self-harm was 2.18% (95% CI 1.99% to 2.36%). For those whose 5 PSs were all in the top 20% of the sample, their combined predicted risk of self-harm was 6.47% (95% CI 6.00% to 6.95%; also see S3 Method, S3 and S4 Tables).

#### Single PS multinomial logistic regressions

S5 Table shows results from 24 multinomial logistic regression tests, using PSs as predictor for 3 possible outcomes: never self-harmed, NSSH, and SSH. When ‘never self-harmed’ was used as the reference group, PSs for bipolar disorder, lifetime cannabis use, and extreme BMI predicted SSH but not NSSH, with *q* < 0.05. However, when NSSH was set as the reference group in order to directly compare NSSH versus SSH, none of the PSs significantly distinguished between NSSH and SSH.

### MR analyses

Table 2 shows the results from MR analyses: ADHD, ALC, bipolar disorder, lifetime cannabis use, MDD, and schizophrenia were exposures in 6 separate univariable MR analyses, with self-harm as the outcome. Out of these 6 exposures, MDD, ADHD, and schizophrenia had MR estimates with *p*-values < 0.05. For the other exposures, none of their MR estimates had *p* < 0.05.

**Table 2:** Univariable and multivariable MR analyses.

Exposure	Method	Univariable MR					Multivariable MR				
		*N*_SNPs_	ß	95% CI lower bound	95% CI upper bound	*p*-Value	*N*_SNPs_	ß	95% CI lower bound	95% CI upper bound	*p*-Value
ADHD	IVW	244	0.003	0.001	0.005	**0.001**	1,206	0.001	−0.001	0.003	0.269
	MR RAPS		0.003	0.001	0.005	**0.001**					
	Weighted median		0.003	2.83 × 10^−4^	0.005	**0.028**					
	MR–Egger regression		0.006	0.001	0.011	**0.031**					
	MR–Egger intercept		−2.57 × 10^−4^	−6.88 × 10^−4^	1.74 × 10^−4^	0.243					
Alcohol dependence disorder	IVW	86	0.001	−4.50 × 10^−4^	0.003	0.157	—	—	—	—	—
	MR RAPS		0.001	−4.57 × 10^−4^	0.003	0.150					
	Weighted median		0.001	−0.001	0.004	0.332					
	MR–Egger regression		0.001	−0.002	0.005	0.465					
	MR–Egger intercept		−1.88 × 10^−5^	−5.85 × 10^−4^	5.48 × 10^−4^	0.948					
Bipolar disorder	IVW	77	0.001	−0.001	0.003	0.310	—	—	—	—	—
	MR RAPS		0.001	−0.001	0.003	0.355					
	Weighted median		1.27 × 10^−4^	−0.002	0.003	0.922					
	MR–Egger regression		3.22 × 10^−4^	−0.008	0.008	0.936					
	MR–Egger intercept		8.31 × 10^−5^	−9.39 × 10^−4^	0.001	0.874					
Lifetime cannabis use	IVW	85	−2.07 × 10^−4^	−0.002	0.001	0.787	—	—	—	—	—
	MR RAPS		−2.85 × 10^−4^	−0.002	0.001	0.730					
	Weighted median		−0.001	−0.004	0.001	0.314					
	MR–Egger regression		−0.002	−0.006	0.001	0.171					
	MR–Egger intercept		3.62 × 10^−4^	−1.38 × 10^−4^	8.61 × 10^−4^	0.160					
MDD	IVW	239	0.008	0.005	0.011	**2.84 × 10**^**−8**^	1,206	0.011	0.007	0.015	**1.04 × 10**^**−12**^
	MR RAPS		0.008	0.005	0.011	**1.24 × 10**^**−7**^					
	Weighted median		0.006	0.001	0.011	**0.013**					
	MR–Egger regression		0.002	−0.004	0.008	0.463					
	MR–Egger intercept		4.20 × 10^−4^	6.06 × 10^−5^	7.80 × 10^−4^	**0.023**					
Schizophrenia	IVW	1,003	0.003	0.002	0.004	**1.54 × 10**^**−9**^	1,206	0.002	4.00 × 10^−5^	0.004	**0.002**
	MR RAPS		0.003	0.002	0.004	**2.89 × 10**^**−9**^					
	Weighted median		0.003	0.002	0.005	**1.80 × 10**^**−5**^					
	MR–Egger regression		0.004	0.001	0.007	**0.009**					
	MR–Egger intercept		−4.84 × 10^−5^	−2.42 × 10^−4^	1.45 × 10^−4^	0.625					

The *p*-values in bold are those that met the *p* < 0.05 threshold. The outcome in these analyses is self-harm.

ADHD, attention deficit/hyperactivity disorder; IVW, inverse variance weighting; MDD, major depressive disorder; MR, Mendelian randomisation; RAPS, robust adjusted profile score.

For MDD, despite having the strongest IVW (β = 0.008, 95% CI 0.005 to 0.011, *p* = 2.84 × 10^−8^), MR RAPS (β = 0.008, 95% CI 0.005 to 0.011, *p* = 1.24 × 10^−7^), and weighted median (β = 0.006, 95% CI 0.001 to 0.011, *p* = 0.013) estimates among the 3 exposures with significant MR estimates, the MR–Egger regression estimate was not significant. For ADHD and schizophrenia, all MR estimates were significant. In all cases, the *I*^2^_GX_ values were >90% (see S6 Table), suggesting that regression dilution has not substantially impacted the MR–Egger analyses [66].

Significance of intercept terms in MR–Egger analyses indicates the presence of pleiotropy. None of the MR–Egger intercept terms were significant, except for MDD (*p* = 0.023). MR Steiger filtering could only be conducted for MDD and schizophrenia (see Text B in S2 Method). Selected SNPs of these 2 exposures are more predictive of the respective exposures than self-harm, suggesting that reverse causation is unlikely to explain our findings. Hence, no SNPs were excluded following the MR Steiger filtering.

When ADHD, MDD, and schizophrenia were included as exposures in multivariable IVW MR analysis, only MDD (β = 0.011, 95% CI 0.007 to 0.015, *p* = 1.04 × 10^−12^) and schizophrenia (β = 0.002, 95% CI 4.00 × 10^−5^ to 0.004, *p* = 0.002) remained as independent predictors of self-harm. Due to the potential presence of pleiotropy between MDD and self-harm, another multivariable IVW MR analysis was conducted with only ADHD and schizophrenia as exposures. Both ADHD (β = 0.003, 95% CI 0.001 to 0.005, *p* = 2.21 × 10^−4^) and schizophrenia (β = 0.003, 95% CI 0.002 to 0.004, *p* = 7.60 × 10^−7^) were significant predictors in this model.

We repeated the multivariable IVW MR analysis using a genome-wide threshold in selecting the instruments for schizophrenia. Similar results were observed, whereby only MDD (β = 0.010, 95% CI 0.006 to 0.014, *p* = 5.48 × 10^−8^) and schizophrenia (β = 0.003, 95% CI 0.001 to 0.005, *p* = 0.002) remained as significant predictors for self-harm (see S7 Table). Results for MR analyses with SSH as the outcome are presented in S8 Table. Only MR estimates for schizophrenia were significant (possibly due to the lower prevalence of SSH, leading to a corresponding loss of power in the SSH GWAS).

**Fig 4:**
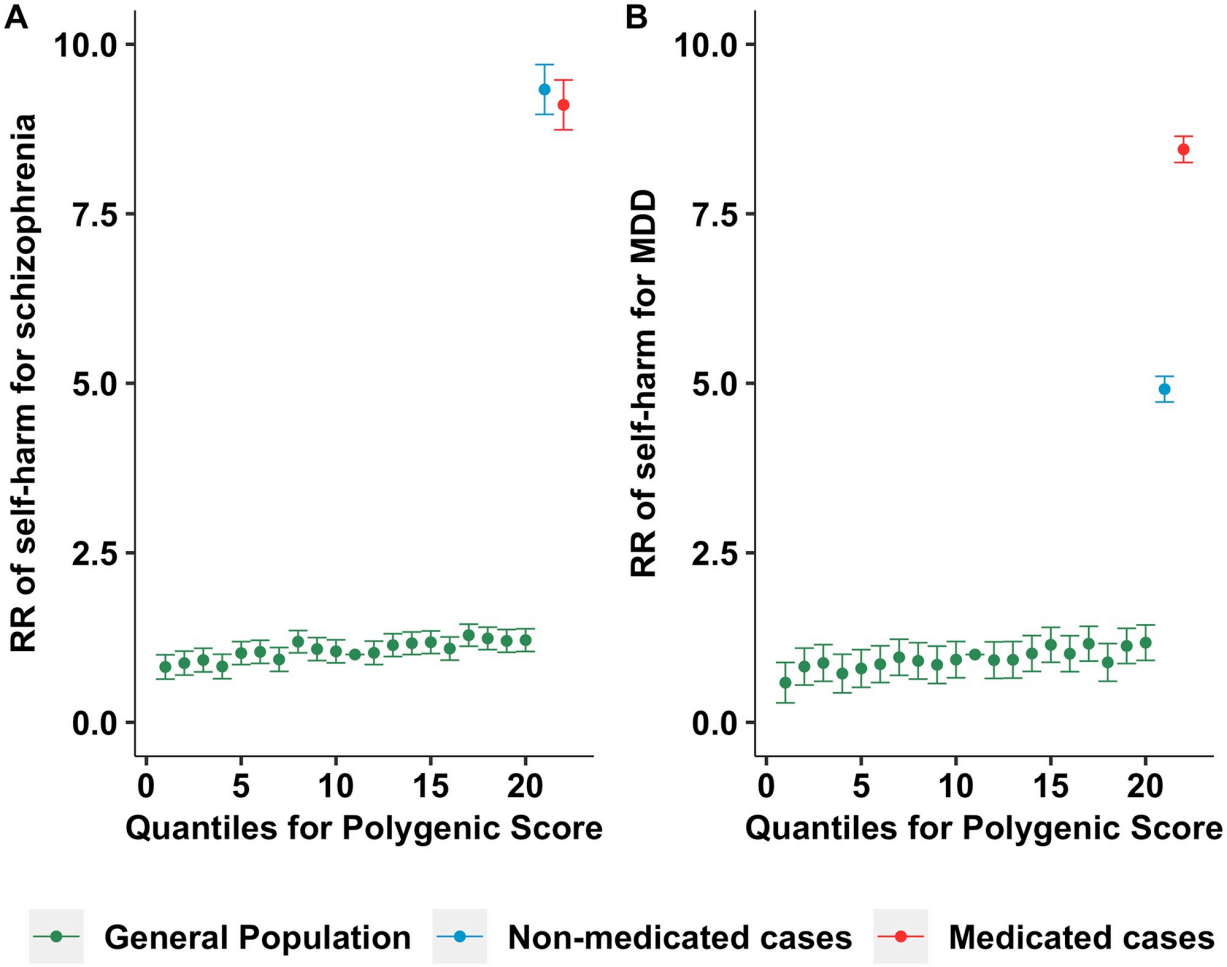
Risk ratios of self-harm in the general population and in non-medicated and medicated MDD and schizophrenia cases. Risk ratios of self-harm in the general population, non-medicated cases, and medicated cases were compared to those with median polygenic score (11th quantile) for schizophrenia (A) and MDD (B). Out of 177 schizophrenia cases in the final analytical sample, 89 (50.3%) of them were medicated. Out of 34,680 MDD cases in the final analytical sample, 7,852 (22.6%) of them were medicated. MDD, major depressive disorder; RR, risk ratio.

In complementary analyses that excluded cases with MDD and schizophrenia diagnoses, PSs for MDD and schizophrenia predicted self-harm in a healthy screened cohort, indicating that genetic liabilities can predict self-harm when the influence of diagnoses is excluded (See Table A in S1 Appendix). As shown in Fig 4, individuals diagnosed with schizophrenia and MDD appear to be at much larger risk for self-harm than the rest of the population. For MDD, medicated cases were at higher risk of self-harm than non-medicated cases, which was not the case for schizophrenia.

## Discussion

We used multiple PSs as genetic proxies to systematically investigate multiple individual vulnerabilities and traits as risk factors for self-harm in a population sample. In PS analyses, we identified 6 risk factors (MDD, schizophrenia, ADHD, bipolar disorder, ALC, and lifetime cannabis use) that predicted self-harm. Five among them (all except for ALC) remained significant in a multiple PS regression. We found little evidence of differential prediction for SSH versus NSSH. In follow-up MR analyses, MDD, schizophrenia, and ADHD emerged as plausible causal risk factors for self-harm, despite evidence of unmediated pleiotropy for MDD.

### Insights into the aetiology of self-harm

Results from our PS methods corroborated previous observational findings where MDD [8], schizophrenia [68], ADHD [69], bipolar disorder [8], and ALC [10] were phenotypically associated with self-harm. Our results are also consistent with positive associations found in PS studies for MDD [22–24], schizophrenia [25], and bipolar disorder [29]. Previous mixed findings for these PSs may have stemmed from lack of power, as sample sizes for those studies varied widely. The current study adds the PSs for ADHD and ALC as novel PSs genetically associated with self-harm. However, when controlling for other PSs, the PS for ALC did not significantly predict self-harm. This finding may suggest that the genetic liability for ALC does not independently predict self-harm when the effects of genetic liability for MDD, bipolar disorder, schizophrenia, ADHD, and lifetime cannabis use are accounted for. For example, ALC may be a marker for a true predictor such as impulsivity, which is more efficiently captured in the PS for ADHD [70]. Alternatively, null findings for ALC may also be due to a lack of power compared to other PSs. Hence, we cannot completely rule out that the PS for ALC has an independent effect on self-harm and there may be a corresponding causal effect of ALC on self-harm.

Most of the PSs that predicted self-harm in this study relate to psychiatric conditions, which confirms the prominence of psychiatric conditions in the aetiology of self-harm [71]. Cognitive traits, physical traits, and personality traits were not found to be associated with self-harm using the PS approach, although previous observational findings found significant phenotypic associations for these domains [12–14]. The absence of significant findings in our study is unlikely to be solely due to lack of power, given that GWASs for some of these traits are more powerful than GWASs for psychiatric conditions (e.g., BMI and education attainment). These findings suggest that these traits and vulnerabilities are unlikely to have (strong) causal effects on self-harm.

Our MR analyses further supported the role of MDD, ADHD, and schizophrenia in the aetiology of self-harm. Psychiatric conditions such as schizophrenia and MDD are among possible stressors in the stress–diathesis model of suicidal behaviour, whereas ADHD, which includes symptoms of impulsivity, may be considered as part of the diathesis in the model [15–18]. Our results showed that traits pertaining to both the stress and diathesis dimensions are contributing to the aetiology of self-harm. An intriguing finding is the presence of significant pleiotropy in the case of MDD. Rather than signifying that MDD does not have a causal effect on self-harm, this may reflect a measurement issue. Indeed, one of the diagnostic criteria for MDD is related to having suicidal thoughts and attempts, which could artificially introduce a pleiotropic effect [7]. To deal with this issue, future studies could rely on a GWAS for MDD excluding the diagnostic criterion related to suicidal thoughts and attempts. This might also explain why, in the multivariable MR analysis, the effect of ADHD was no longer significant—as we partially controlled for self-harm—whereas it was significant when considering only ADHD and schizophrenia.

The current study found mixed results for whether there are distinct aetiologies for SSH and NSSH. Most PSs that predicted self-harm also predicted both SSH and NSSH, except the PSs for bipolar disorder, lifetime cannabis use, and extreme BMI, which only predicted SSH but not NSSH. However, in a formal test comparing NSSH and SSH, the estimates of these 3 risk factors were not significantly different between NSSH and SSH. Hence, our findings do not provide evidence for marked differences in aetiology between SSH and NSSH.

### Clinical implications

This study suggests that individual vulnerabilities and traits underlying self-harm most likely relate to psychiatric conditions (i.e., MDD and schizophrenia), rather than other domains such as personality traits. Hence, treatments focusing on the core symptoms of these psychiatric conditions are important in preventing or addressing the risk of self-harm. Findings from PS analyses suggest that genetic liabilities for these conditions increase the likelihood of self-harm even in those not clinically diagnosed, suggesting that subthreshold symptoms of these psychiatric conditions may increase the risk of self-harm. Clinicians may want to systematically test for such symptoms in self-harming patients. Future investigations could test whether drugs for such core conditions can be repurposed for treating self-harming patients with either full-blown or subthreshold conditions. For example, prescription of methylphenidate for ADHD treatment was found to be associated with reduction of suicide risk [72]. As a note of caution, medicated schizophrenia cases were not at less risk of self-harm than non-medicated patients, whereas medicated MDD patients were at substantially higher risk for self-harm in the present sample. This could be due to medicated patients having more severe symptoms than non-medicated patients, or to adverse effects of medication, particularly for MDD, where suicidality might be an adverse effect of antidepressant treatment [73].

### Limitations

To avoid the overlapping of discovery and target samples, we excluded GWASs that contain the UK Biobank sample, resulting in our selecting older GWASs for generating PSs in some cases. This might have led to non-significant findings due to lack of power. The results should be generalised with caution because the UK Biobank is not representative of the UK population, as participants are more educated, older, wealthier, and healthier [74]. The questions asked in the mental health questionnaire were retrospective and their formulation led to an exclusive dichotomy between NSSH or SSH, although some might have engaged in both NSSH and SSH at different times.

PSs in this study explained only a small amount of the variance in self-harm. The maximum variance explained by any single PS was 0.4%, for the MDD PS. Importantly, such prediction of self-harm is likely indirect, i.e., we expect the PS for a given risk factor to influence this risk factor first, and then self-harm only to the extent that this risk factor is a causal risk for self-harm. As a result, given that, for example, the PS for MDD only explains 1.9% of the variance in the liability for MDD [37], it is unsurprising that its effect on self-harm is much lower. In addition, if the PS for MDD were to capture the full heritability of MDD (which is 37%, according to a meta-analysis of twin studies [75]), the explanatory power of the MDD PS could be expected to proportionally increase up to 7.8%. In turn, this would contribute to explaining the gap between the currently estimated heritability of self-harm in family-based studies (up to about 50% [76]) and the low percentage of variance explained in our analyses. Any remaining gap would indicate that other genetically influenced risk factors for self-harm remain to be identified beyond those uncovered in the present study.

The lack of evidence showing significant aetiological differences between NSSH and SSH may also be due to lack of power for subset analyses. However, the effect sizes of the PSs for both NSSH and SSH appeared similar overall, suggesting that the aetiology of NSSH and SSH may be similar. For MR analyses, although a range of sensitivity analyses were conducted to account for pleiotropy and weak instrument bias, they cannot completely rule out horizontal pleiotropy. The possibility thus remains that significant associations are non-causal. On the other hand, risk factors that were non-significant in MR analyses should not be entirely ruled out as potential causal risk factors for self-harm. Future studies with more powerful genetic instruments may uncover new risk factors that we were unable to identify.

### Conclusion

Among 24 PSs used as genetic proxies for vulnerabilities and traits possibly associated with self-harm, we found that PSs for MDD, schizophrenia, ADHD, bipolar disorder, ALC, and lifetime cannabis use were statistically significant. After a series of complementary analyses to further strengthen causal inference, schizophrenia emerged as the most plausible causal risk factor, followed by MDD and ADHD. Detection and treatment of core symptoms of these conditions, such as psychotic or impulsivity symptoms, may benefit self-harming patients.

## Supporting information

S1 Appendix Complementary analyses. (DOCX)

S1 Checklist STROBE checklist. (DOCX)

S1 Fig Pseudo *R*^2^ plots of 6 PSs in predicting self-harm. (DOCX)

S1 Method Genotyping, imputation, and quality control. (DOCX)

S2 Method Mendelian randomisation. (DOCX)

S3 Method Predicted risk of self-harm. (DOCX)

S1 Table Descriptive statistics for each group of self-harm-related phenotypes. (DOCX)

S2 Table Correlations between PSs that were significant in single PS regression in predicting self-harm. (DOCX)

S3 Table Single PS prediction of self-harm risk. (DOCX)

S4 Table Multiple PS prediction of self-harm risk. (DOCX)

S5 Table Multinomial regression models in predicting SSH and NSSH. (DOCX)

S6 Table *I*2GX statistics for the MR instruments in univariable MR analyses. (DOCX)

S7 Table Multivariable MR with self-harm as outcome, using a genome-wide *p*-value threshold (*p* < 5 × 10^−8^) to select the instruments. (DOCX)

S8 Table Univariable MR results with SSH as the outcome. (DOCX)

## Abbreviations

ADHD: attention deficit/hyperactivity disorder
ALC: alcohol dependence disorder
FDR: false discovery rate
GWAS: genome-wide association study
IVW: inverse variance weighting
MDD: major depressive disorder
MR: Mendelian randomisation
NSSH: non-suicidal self-harm
OR: odds ratio
PS: polygenic score
RAPS: robust adjusted profile score
SSH: suicidal self-harm
