# Supplementary material for: Studying individual risk factors for self-harm in the UK Biobank: A polygenic scoring and Mendelian randomisation study
Source: PLoS Med. 2020 Jun 1;17(6):e1003137. doi: 10.1371/journal.pmed.1003137 (PMC7263593; doi:10.1371/journal.pmed.1003137)
Supplement: S1 Appendix — (DOCX) [file pmed.1003137.s001.docx]

**S1 Appendix. Complementary analyses**

In complementary analyses, single PS binomial regression tests were repeated for these risk factors by excluding cases who are associated with these risk factors (i.e. having diagnoses for MDD or schizophrenia). This was to test if the association between their respective PS and self-harm remains after those with the respective psychiatric disorder diagnoses were excluded. Results are shown in Table A.

**Definition of cases**

Cases for MDD and schizophrenia were identified respectively from UK Biobank’s primary and secondary ICD 9 or ICD 10 diagnoses, non-cancer illness self-report, and MHQ self-report. The details are shown in the table below. Number of cases derived from each data field are presented in Figures A (for MDD) and B (for schizophrenia).

| Phenotype | Data field |
| --- | --- |
| ICD 9 diagnosis (primary) | 41203 |
| ICD 9 diagnosis (secondary) | 41205 |
| ICD 10 diagnosis (primary) | 41202 |
| ICD 10 diagnosis (secondary) | 41204 |
| Non-cancer illness self-report | 20002 |
| MHQ self-report (“Have you been diagnosed with one or more of the following mental health problems by a professional, even if you don't have it currently?”) | 20544 |


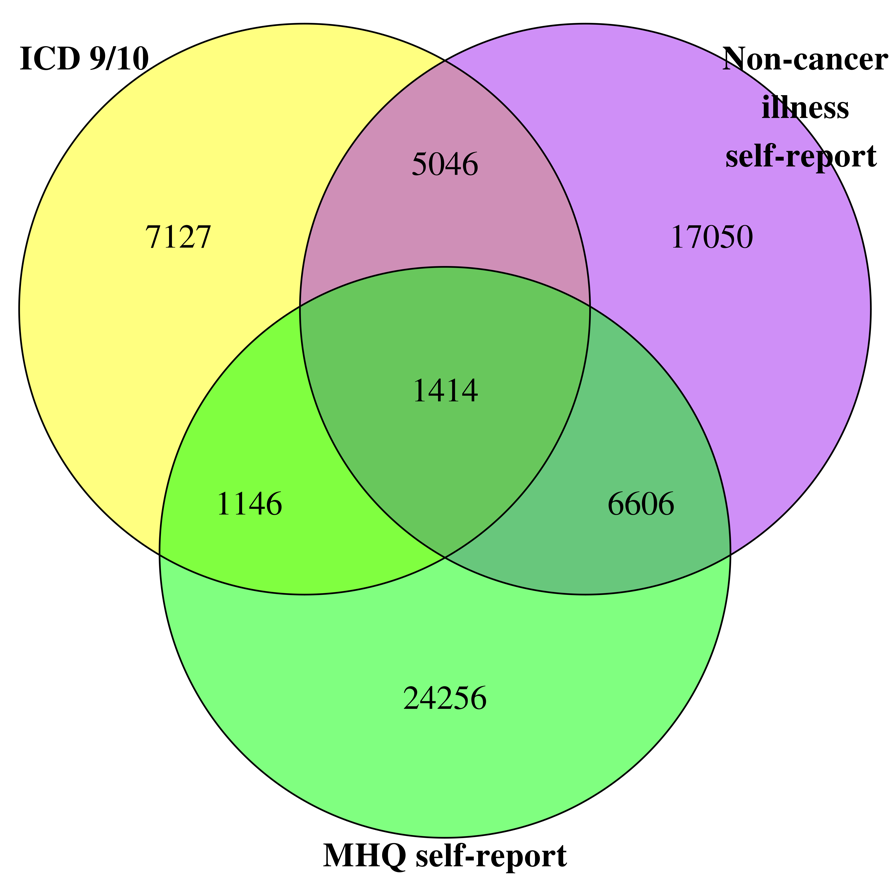


**Figure A. Number of MDD cases derived from respective data fields. Total=62,645 cases.**

## **
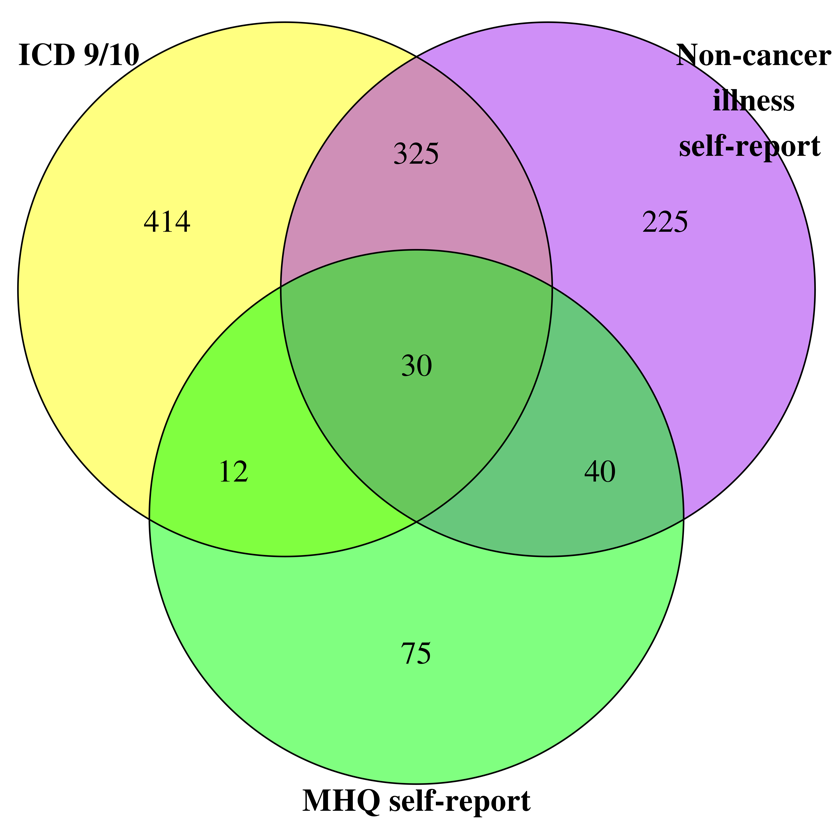
**

**Figure B. Number of schizophrenia cases derived from respective data fields. Total=1,121 cases.**

***Definition of medication***

Lists of antidepressants and antipsychotics prescribed for MDD and schizophrenia patients were compiled from the Mind website (for antidepressants: <https://www.mind.org.uk/information-support/drugs-and-treatments/antidepressants-a-z/#.XP-iKy3Mw1I>; for antipsychotics: <https://www.mind.org.uk/information-support/drugs-and-treatments/antipsychotics-a-z/overview/?o=60249#.XP-iUS3Mw1I> ). We also visited British National Formulary (BNF) website ([https://www.bnf.org](https://www.bnf.org/)) to identify other medications used for MDD or schizophrenia. Data of medications taken by the participants were derived from UK Biobank’s treatment/medication code (data field 20003).

***Calculation of risk ratios in Fig 4***

For MDD, we separate the participants into three main groups: medicated cases, non-medicated cases, and general population. For general population, the participants were further subdivided into 20 quantiles according to their polygenic score for MDD. Risk ratios of self-harm for all groups compared with those with the median polygenic score (the 11^th^ quantile) were derived using Poisson regression. These are repeated for schizophrenia. Results are shown in Fig 4 and Table B in this appendix.

**Results of complementary analyses**

**Table A. Single PS binomial regressions predicting self-harm with cases excluded.**

| **PS (with cases removed)** | **OR** | **95% CI** | ***p*-value** |
| --- | --- | --- | --- |
| Schizophrenia | 1.131 | 1.099, 1.164 | 6.78E-17 |
| MDD | 1.148 | 1.097, 1.201 | 1.96E-09 |

**Table B. Risk ratios of self-harm in the general population and medicated and non-medicated MDD and schizophrenia cases.**

| Quantile | MDD | | | Schizophrenia | | |
| --- | --- | --- | --- | --- | --- | --- |
|  | Risk ratios | 95% CI  lower bound | 95% CI  upper bound | Risk ratios | 95% CI  lower bound | 95% CI  upper bound |
| 1^st^ quantile | 0.59 | 0.29 | 0.88 | 0.82 | 0.64 | 1.00 |
| 2^nd^ quantile | 0.82 | 0.55 | 1.09 | 0.87 | 0.70 | 1.05 |
| 3^rd^ quantile | 0.88 | 0.61 | 1.15 | 0.92 | 0.74 | 1.09 |
| 4^th^ quantile | 0.72 | 0.43 | 1.00 | 0.82 | 0.64 | 1.00 |
| 5^th^ quantile | 0.79 | 0.52 | 1.07 | 1.02 | 0.85 | 1.19 |
| 6^th^ quantile | 0.86 | 0.59 | 1.13 | 1.04 | 0.87 | 1.21 |
| 7^th^ quantile | 0.96 | 0.69 | 1.23 | 0.93 | 0.75 | 1.10 |
| 8^th^ quantile | 0.91 | 0.64 | 1.17 | 1.19 | 1.02 | 1.35 |
| 9^th^ quantile | 0.85 | 0.57 | 1.12 | 1.08 | 0.91 | 1.25 |
| 10^th^ quantile | 0.92 | 0.66 | 1.19 | 1.05 | 0.88 | 1.22 |
| 11^th^ quantile (reference group) | 1.00 | 1.00 | 1.00 | 1.00 | 1.00 | 1.00 |
| 12^th^ quantile | 0.92 | 0.65 | 1.19 | 1.02 | 0.85 | 1.20 |
| 13^th^ quantile | 0.92 | 0.65 | 1.19 | 1.14 | 0.97 | 1.31 |
| 14^th^ quantile | 1.02 | 0.75 | 1.28 | 1.17 | 1.00 | 1.33 |
| 15^th^ quantile | 1.14 | 0.89 | 1.40 | 1.18 | 1.01 | 1.35 |
| 16^th^ quantile | 1.01 | 0.75 | 1.28 | 1.09 | 0.92 | 1.26 |
| 17^th^ quantile | 1.16 | 0.90 | 1.42 | 1.28 | 1.12 | 1.45 |
| 18^th^ quantile | 0.88 | 0.61 | 1.16 | 1.24 | 1.07 | 1.40 |
| 19^th^ quantile | 1.13 | 0.87 | 1.39 | 1.20 | 1.03 | 1.37 |
| 20^th^ quantile | 1.17 | 0.91 | 1.44 | 1.21 | 1.05 | 1.38 |
| Non-medicated cases | 4.91 | 4.72 | 5.10 | 9.33 | 8.97 | 9.70 |
| Medicated cases | 8.45 | 8.26 | 8.64 | 9.11 | 8.74 | 9.47 |
