# Supplementary material for: Studying individual risk factors for self-harm in the UK Biobank: A polygenic scoring and Mendelian randomisation study
Source: PLoS Med. 2020 Jun 1;17(6):e1003137. doi: 10.1371/journal.pmed.1003137 (PMC7263593; doi:10.1371/journal.pmed.1003137)
Supplement: S1 Checklist — (DOCX) [file pmed.1003137.s002.docx]

**S1 Checklist. STROBE checklist**

(downloaded and adapted from <https://www.strobe-statement.org/index.php?id=available-checklists>)

**Manuscript title : Studying Individual Risk Factors for Self-Harm in the UK Biobank: A Polygenic Scoring and Mendelian Randomization Study**

|  | Item |  | Section |
| --- | --- | --- | --- |
| **Title and abstract** | 1 | (a) Indicate the study’s design with a commonly used term in the title or the abstract | Title |
|  |  | (b) Provide in the abstract an informative and balanced summary of what was done and what was found | Abstract |
|  |  |  |  |
| **Introduction** |  |  |  |
| Background/rational | 2 | Explain the scientific background and rationale for the investigation being reported | Introduction, paragraphs 1-5 |
| Objectives | 3 | State specific objectives, including any prespecified hypotheses | Introduction, paragraph 6 |
|  |  |  |  |
| **Methods** |  |  |  |
| Study design | 4 | Present key elements of study design early in the paper | Introduction, paragraph 6 |
| Setting | 5 | Describe the setting, locations, and relevant dates, including periods of recruitment, exposure, follow-up, and data collection | Methods, participants section |
| Participants | 6 | (a) Cohort study—Give the eligibility criteria, and the sources and methods of selection of participants. Describe methods of follow-up Case-control study—Give the eligibility criteria, and the sources and methods of case ascertainment and control selection. Give the rationale for the choice of cases and controls Cross-sectional study—Give the eligibility criteria, and the sources and methods of selection of participants | Methods, participants section and Fig 1 |
|  |  | (b) Cohort study—For matched studies, give matching criteria and number of exposed and unexposed Case-control study—For matched studies, give matching criteria and the number of controls per case | Fig 1 |
| Variables | 7 | Clearly define all outcomes, exposures, predictors, potential confounders, and effect modifiers. Give diagnostic criteria, if applicable | Methods, “Defining self-harm phenotypes” section and Table 1 |
| Data sources/ measurement | 8 | For each variable of interest, give sources of data and details of methods of assessment (measurement). Describe comparability of assessment methods if there is more than one group | Methods, participants section and “defining self-harm phenotypes” section |
| Bias | 9 | Describe any efforts to address potential sources of bias | Methods, under MR analyses section |
| Study size | 10 | Explain how the study size was arrived at | Fig 1 and methods, participants section |
| Quantitative variables | 11 | Explain how quantitative variables were handled in the analyses. If applicable, describe which groupings were chosen and why | Methods, statistical analyses section |
| Statistical methods | 12 | (a) Describe all statistical methods, including those used to control for confounding | Methods, statistical analyses section |
|  |  | (b) Describe any methods used to examine subgroups and interactions | S1 Supporting Information |
|  |  | (c) Explain how missing data were addressed | Methods, “Defining self-harm phenotypes” section |
|  |  | (d) Cohort study—If applicable, explain how loss to follow-up was addressed Case-control study—If applicable, explain how matching of cases and controls was addressed Cross-sectional study—If applicable, describe analytical methods taking account of sampling strategy | Methods, “Defining self-harm phenotypes” section |
|  |  | (e) Describe any sensitivity analyses | Methods, under MR analyses section, and S1 Supporting information |
|  |  |  |  |
| **Results** |  |  |  |
| Participants | 13 | (a) Report numbers of individuals at each stage of study—eg numbers potentially eligible, examined for eligibility, confirmed eligible, included in the study, completing follow-up, and analysed | Fig 1 |
|  |  | (b) Give reasons for non-participation at each stage | Fig 1 |
|  |  | (c) Consider use of a flow diagram | Fig 1 |
| Descriptive data | 14 | (a) Give characteristics of study participants (eg demographic, clinical, social) and information on exposures and potential confounders | S1 Table |
|  |  | (b) Indicate number of participants with missing data for each variable of interest | Fig 1 |
|  |  | (c) Cohort study—Summarise follow-up time (eg, average and total amount) | N/A |
| Outcome data | 15 | Cohort study—Report numbers of outcome events or summary measures over time Case-control study—Report numbers in each exposure category, or summary measures of exposure Cross-sectional study—Report numbers of outcome events or summary measures | Fig 1 |
| Main results | 16 | (a) Give unadjusted estimates and, if applicable, confounder-adjusted estimates and their precision (eg, 95% confidence interval). Make clear which confounders were adjusted for and why they were included | Table 1 and Table 2 |
|  |  | (b) Report category boundaries when continuous variables were categorized | N/A |
|  |  | (c) If relevant, consider translating estimates of relative risk into absolute risk for a meaningful time period | N/A |
| Other analyses | 17 | Report other analyses done—eg analyses of subgroups and interactions, and sensitivity analyses | Fig 4 and S1 Supporting Information |
|  |  |  |  |
| **Discussion** |  |  |  |
| Key results | 18 | Summarise key results with reference to study objectives | Discussion, paragraphs 1 and 5 |
| Limitations | 19 | Discuss limitations of the study, taking into account sources of potential bias or imprecision. Discuss both direction and magnitude of any potential bias | Discussion, limitation section |
| Interpretation | 20 | Give a cautious overall interpretation of results considering objectives, limitations, multiplicity of analyses, results from similar studies, and other relevant evidence | Discussion, paragraphs 2,3,4 and 6 |
| Generalisability | 21 | Discuss the generalisability (external validity) of the study results | Discussion, limitation section |
|  |  |  |  |
| **Other information** |  |  |  |
| Funding | 22 | Give the source of funding and the role of the funders for the present study and, if applicable, for the original study on which the present article is based | Given on article submission page |
