# Supplementary figures and images for: Studying individual risk factors for self-harm in the UK Biobank: A polygenic scoring and Mendelian randomisation study
Source: PLoS Med. 2020 Jun 1;17(6):e1003137. doi: 10.1371/journal.pmed.1003137 (PMC7263593; doi:10.1371/journal.pmed.1003137)

**S1 Figure. Pseudo R2 plots of 6 PSs in predicting self-harm.**


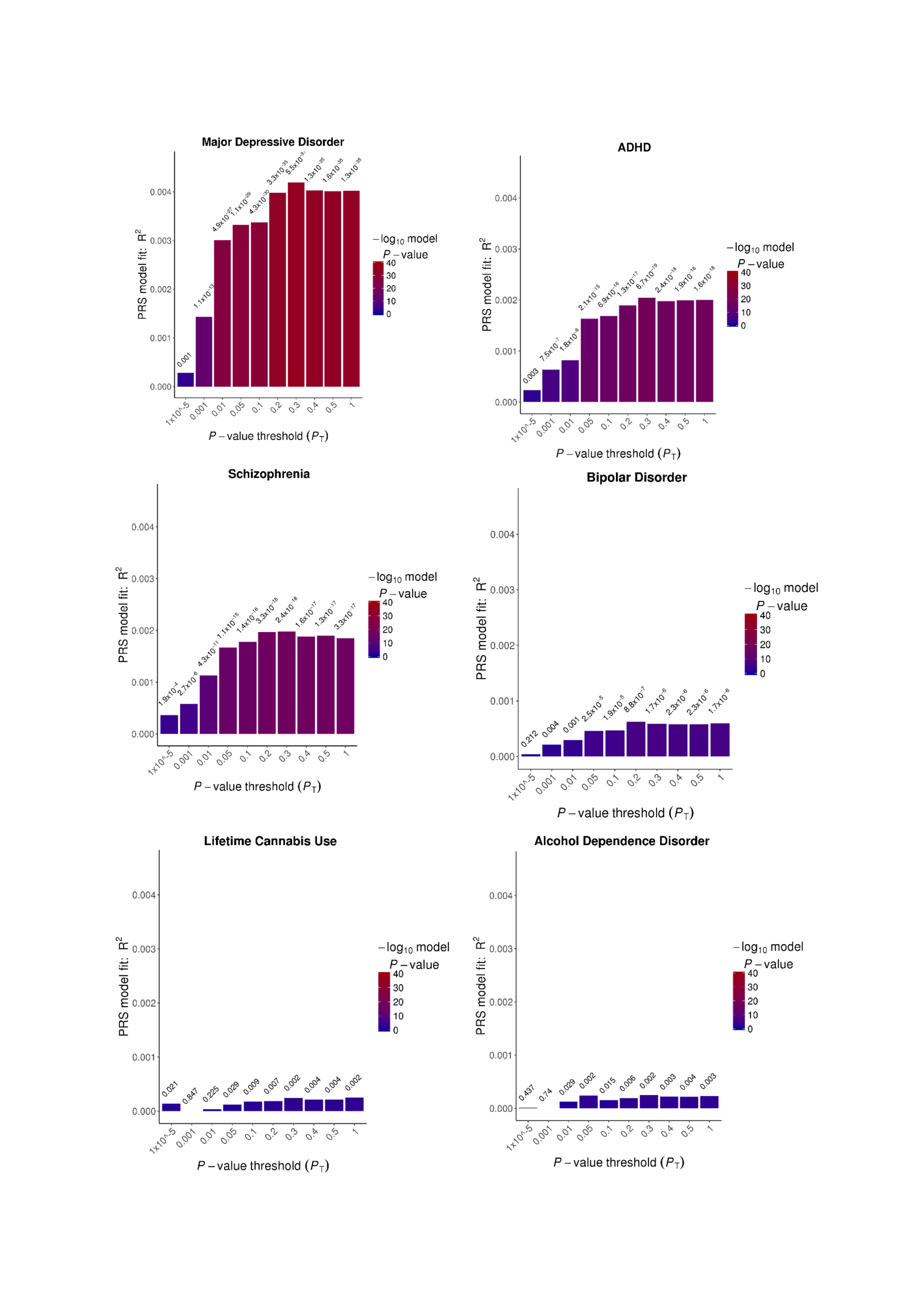

Supplement: S1 Fig — (DOCX) [file pmed.1003137.s003.docx]
