# Supplementary material for: Studying individual risk factors for self-harm in the UK Biobank: A polygenic scoring and Mendelian randomisation study
Source: PLoS Med. 2020 Jun 1;17(6):e1003137. doi: 10.1371/journal.pmed.1003137 (PMC7263593; doi:10.1371/journal.pmed.1003137)
Supplement: S1 Method — (DOCX) [file pmed.1003137.s004.docx]

**S1 Method. Genotyping, imputation and quality control**

Participants were genotyped across 22 assessment centres in the UK, using either the Affymetrix UK BiLEVE Axiom array or the Affymetrix UK Biobank Axiom® array [1]. These two arrays share over 95% common content. Further details of genotyping and sample processing are available (<http://biobank.ctsu.ox.ac.uk/crystal/docs/genotyping_affy_sampro.pdf>). Before the release of UK Biobank genetic data, a stringent quality control (QC) protocol was performed, with further imputation [1] using the Haplotype Reference Consortium (HRC) reference panel [2]. Details of the QC protocol (<http://biobank.ctsu.ox.ac.uk/crystal/docs/genotyping_qc.pdf>) and genetic imputation (<http://www.ukbiobank.ac.uk/wp-content/uploads/2014/04/imputation_documentation_May2015.pdf>) are available online.

Prior to the analyses in the current study, further QC steps were taken. Participants were excluded if they were reported as outliers based on heterozygosity and missing rates, had a variant call rate < 98%, had a mismatch of phenotypic and genotypic gender, or had non-European ancestry as defined by 4-means clustering of the first two PCs from the genetic data. By using the KING toolset [3], one of each pair of participants sharing relatedness of up to the third degree (KING estimated kinship coefficient > 0.044) were also excluded. Removal of relatives was performed using a "greedy" algorithm, which minimises exclusions (for example, by excluding the child in a mother-father-child trio). Genetic variants were excluded if they had a call rate < 98%, a minor allele frequency < 0.01, or deviated from the Hardy-Weinberg equilibrium (*p* < 10^-8^). Principal components analysis was also performed on the European-only subset of the data using the software flashpca2 [4].

References

1. Bycroft C, Freeman C, Petkova D, Band G, Elliott LT, Sharp K, et al. The UK Biobank resource with deep phenotyping and genomic data. Nature. 2018;562: 203–209. doi:10.1038/s41586-018-0579-z

2. McCarthy S, Das S, Kretzschmar W, Delaneau O, Wood AR, Teumer A, et al. A reference panel of 64,976 haplotypes for genotype imputation. Nat Genet. 2016;48: 1279–1283. doi:10.1038/ng.3643

3. Manichaikul A, Mychaleckyj JC, Rich SS, Daly K, Sale M, Chen W-M. Robust relationship inference in genome-wide association studies. Bioinformatics. 2010;26: 2867–2873. doi:10.1093/bioinformatics/btq559

4. Abraham G, Qiu Y, Inouye M. FlashPCA2: principal component analysis of Biobank-scale genotype datasets. Stegle O, editor. Bioinformatics. 2017;33: 2776–2778. doi:10.1093/bioinformatics/btx299
