# Supplementary material for: Studying individual risk factors for self-harm in the UK Biobank: A polygenic scoring and Mendelian randomisation study
Source: PLoS Med. 2020 Jun 1;17(6):e1003137. doi: 10.1371/journal.pmed.1003137 (PMC7263593; doi:10.1371/journal.pmed.1003137)
Supplement: S2 Method — (DOCX) [file pmed.1003137.s005.docx]

**S2 Method. Mendelian randomisation**

**Text A: MR clumping and harmonization**

Clumping of SNPs with r^2^ < ·001 within 250 kb was applied. SNPs in exposures and outcomes were harmonized by flipping alleles where possible, using allele frequencies to infer strands of ambiguous SNPs. Non-inferable SNPs with minor allele frequency > 0·42 were discarded.

**Text B: MR Steiger filtering**

MR Steiger filtering was implemented to address the possibility of reverse causation [1]. For each SNP, we expect that the effect size for the association with the exposure should be larger than the effect size for the association with the outcome. This is because the effect on the outcome is hypothesised to be indirect through the exposure. As such, all SNPs for which the effect size of the association with the outcome was larger than the one with the exposure were filtered out before reimplementing MR.

In this study, MR Steiger filtering could only be applied to test the direction of causality between MDD and schizophrenia with self-harm because the summary statistics for other exposures did not contain information about allele frequencies, which are needed for the test.

For MDD, out of the 239 SNPs, we found that all SNPs were in the correct direction (i.e. larger association with MDD than with self-harm). For schizophrenia, all 1003 SNPs were also in the correct direction. Hence, no SNP was filtered out.

**Reference**

1. Hemani G, Tilling K, Davey Smith G. Orienting the causal relationship between imprecisely measured traits using GWAS summary data. PLoS Genet. 2017;13: e1007081. doi:10.1371/journal.pgen.1007081
