# Supplementary material for: Studying individual risk factors for self-harm in the UK Biobank: A polygenic scoring and Mendelian randomisation study
Source: PLoS Med. 2020 Jun 1;17(6):e1003137. doi: 10.1371/journal.pmed.1003137 (PMC7263593; doi:10.1371/journal.pmed.1003137)
Supplement: S3 Method — (DOCX) [file pmed.1003137.s006.docx]

**S3 Method: Predicted risk of self-harm**

Multivariate logistic regression was used to model the association between risk of self-harm and five PS (ADHD, bipolar disorder, lifetime cannabis use, MDD and schizophrenia). Age and the first six principal components were included as covariates. The logistic regression model coefficients were used to derive the predicted probabilities of (i) each of the five PS in separation and (ii) the combination of the five PS. We computed the sample mean per quintile for each of the PS and used this as the new dataset for prediction. To estimate the predicted probability of each of the five PS in separation, we included only the data of the PS of interest (i.e. mean per quintile), while holding all other variables in the equation constant (i.e. at their mean value). To estimate the predicted probability of the combination of the five PS, we included the means per quintile for all five PS, keeping only the covariates constant (age and principal components). To assess whether the inclusion of sex alters the results, we also tested a set of models stratified by sex. All analyses were carried out in R version 3.5.0 [1], using the ‘predict’ function and ggplot2 [2] to visualize the results.

References

1. R Core Team. R: A language and environment for statistical computing. Vienna, Austria: R Foundation for Statistical Computing; 2017. Available: https://www.r-project.org/

2. Wickham H. ggplot2: Elegant Graphics for Data Analysis. Springer-Verlag New York; 2016.
