# Supplementary material for: Studying individual risk factors for self-harm in the UK Biobank: A polygenic scoring and Mendelian randomisation study
Source: PLoS Med. 2020 Jun 1;17(6):e1003137. doi: 10.1371/journal.pmed.1003137 (PMC7263593; doi:10.1371/journal.pmed.1003137)
Supplement: S1 Table — (DOCX) [file pmed.1003137.s007.docx]

**S1 Table. Descriptive statistics for each group of self-harm-related phenotypes.**

| Subgroup of sample | Female (%) | Mean age (years) | SD of age (years) |
| --- | --- | --- | --- |
| Full analytical sample | 56·2 | 65·9 | 7·7 |
| Self-harmed | 69·4 | 62·3 | 7·5 |
| SSH | 68·0 | 63·1 | 7·4 |
| NSSH | 70·5 | 61·4 | 7·5 |
| Never self-harmed | 55·6 | 66·1 | 7·7 |
|  |  |  |  |

Note. Descriptive statistics of self-harmed, SSH, NSSH and never self-harmed are based on groups within the full analytical sample.
