# Supplementary material for: Studying individual risk factors for self-harm in the UK Biobank: A polygenic scoring and Mendelian randomisation study
Source: PLoS Med. 2020 Jun 1;17(6):e1003137. doi: 10.1371/journal.pmed.1003137 (PMC7263593; doi:10.1371/journal.pmed.1003137)
Supplement: S2 Table — (DOCX) [file pmed.1003137.s008.docx]

**S2 Table. Correlations between PSs that were significant in single PS regression in predicting self-harm.**

|  | **ADHD** | **ALC** | **Bipolar disorder** | **Lifetime Cannabis use** | **MDD** | **Schizophrenia** |
| --- | --- | --- | --- | --- | --- | --- |
| **ADHD** | 1.00 | 0·05 | 0·01 | 0·02 | 0·21 | 0·06 |
| **ADD** | 0·05 | 1.00 | 0·05 | 0·07 | 0·06 | 0·08 |
| **Bipolar disorder** | 0·01 | 0·05 | 1.00 | 0·04 | 0·10 | 0·22 |
| **Lifetime Cannabis use** | 0·02 | 0·07 | 0·04 | 1.00 | 0·04 | 0·08 |
| **MDD** | 0·21 | 0·06 | 0·10 | 0·04 | 1.00 | 0·16 |
| **Schizophrenia** | 0·06 | 0·08 | 0·22 | 0·08 | 0·16 | 1.00 |

Note. ADHD = Attention-deficit/hyperactivity disorder, ALC = Alcohol dependence disorder, MDD = major depressive disorder.
