# Supplementary material for: Studying individual risk factors for self-harm in the UK Biobank: A polygenic scoring and Mendelian randomisation study
Source: PLoS Med. 2020 Jun 1;17(6):e1003137. doi: 10.1371/journal.pmed.1003137 (PMC7263593; doi:10.1371/journal.pmed.1003137)
Supplement: S3 Table — (DOCX) [file pmed.1003137.s009.docx]

**S3 Table. Single PS prediction of self-harm risk.**

| **Model** | **Quintile** | **Predicted risk (%)** | **95% CI lower bound (%)** | **95% CI upper bound (%)** |
| --- | --- | --- | --- | --- |
| **ADHD** | 1 | 3.334 | 3.171 | 3.498 |
|  | 2 | 3.599 | 3.478 | 3.720 |
|  | 3 | 3.771 | 3.659 | 3.884 |
|  | 4 | 3.953 | 3.824 | 4.081 |
|  | 5 | 4.266 | 4.066 | 4.466 |
| **Bipolar disorder** | 1 | 3.605 | 3.427 | 3.783 |
|  | 2 | 3.708 | 3.583 | 3.832 |
|  | 3 | 3.772 | 3.660 | 3.884 |
|  | 4 | 3.837 | 3.712 | 3.963 |
|  | 5 | 3.947 | 3.759 | 4.135 |
| **Lifetime cannabis use** | 1 | 3.585 | 3.411 | 3.759 |
|  | 2 | 3.700 | 3.576 | 3.823 |
|  | 3 | 3.772 | 3.659 | 3.884 |
|  | 4 | 3.846 | 3.720 | 3.971 |
|  | 5 | 3.969 | 3.781 | 4.157 |
| **MDD** | 1 | 3.104 | 2.945 | 3.262 |
|  | 2 | 3.502 | 3.381 | 3.623 |
|  | 3 | 3.772 | 3.660 | 3.885 |
|  | 4 | 4.061 | 3.932 | 4.190 |
|  | 5 | 4.579 | 4.369 | 4.789 |
| **Schizophrenia** | 1 | 3.306 | 3.136 | 3.475 |
|  | 2 | 3.587 | 3.465 | 3.710 |
|  | 3 | 3.771 | 3.659 | 3.884 |
|  | 4 | 3.964 | 3.834 | 4.093 |
|  | 5 | 4.305 | 4.096 | 4.514 |

Note. Predicted % derived from a multivariable logistic regression model that included all five PS. Covariates included were age and first 6 principle components.
