# Supplementary material for: Studying individual risk factors for self-harm in the UK Biobank: A polygenic scoring and Mendelian randomisation study
Source: PLoS Med. 2020 Jun 1;17(6):e1003137. doi: 10.1371/journal.pmed.1003137 (PMC7263593; doi:10.1371/journal.pmed.1003137)
Supplement: S4 Table — (DOCX) [file pmed.1003137.s010.docx]

**S4 Table. Multiple PS prediction of self-harm risk.**

| **Model** | **Quintile** | **Predicted risk (%)** | **95% CI lower bound (%)** | **95% CI upper bound (%)** |
| --- | --- | --- | --- | --- |
| **Whole sample** | 1 | 2.176 | 1.993 | 2.359 |
|  | 2 | 3.062 | 2.928 | 3.196 |
|  | 3 | 3.770 | 3.658 | 3.883 |
|  | 4 | 4.634 | 4.459 | 4.810 |
|  | 5 | 6.471 | 5.994 | 6.947 |
| **Male sample** | 1 | 1.590 | 1.443 | 1.738 |
|  | 2 | 2.231 | 2.100 | 2.362 |
|  | 3 | 2.744 | 2.609 | 2.878 |
|  | 4 | 3.370 | 3.186 | 3.553 |
|  | 5 | 4.705 | 4.307 | 5.102 |
| **Female sample** | 1 | 2.672 | 2.443 | 2.902 |
|  | 2 | 3.733 | 3.557 | 3.909 |
|  | 3 | 4.574 | 4.415 | 4.733 |
|  | 4 | 5.594 | 5.362 | 5.826 |
|  | 5 | 7.739 | 7.161 | 8.318 |

Note. Predicted % derived from a multivariable logistic regression model that included all five PS. Covariates included were age and first 6 principle components.
