# Supplementary material for: Studying individual risk factors for self-harm in the UK Biobank: A polygenic scoring and Mendelian randomisation study
Source: PLoS Med. 2020 Jun 1;17(6):e1003137. doi: 10.1371/journal.pmed.1003137 (PMC7263593; doi:10.1371/journal.pmed.1003137)
Supplement: S5 Table — (DOCX) [file pmed.1003137.s011.docx]

**S5 Table. Single PS multinomial regression models in predicting SSH and NSSH.**

| **PS of risk factors** | **NSSH vs never self-harmed^a^** | | | | | **SSH vs never self-harmed^b^** | | | | | **NSSH vs SSH^c^** | | | | |
| --- | --- | --- | --- | --- | --- | --- | --- | --- | --- | --- | --- | --- | --- | --- | --- |
|  | **OR** | **95% CI lower bound** | **95% CI upper bound** | **p-value** | **q-value** | **OR** | **95% CI lower bound** | **95% CI upper bound** | **p-value** | **q-value** | **OR** | **95% CI lower bound** | **95% CI upper bound** | **p-value** | **q-value** |
| **Mental Health Vulnerabilities** |  |  |  |  |  |  |  |  |  |  |  |  |  |  |  |
| ADHD symptoms | 1·043 | 1·043 | 1·002 | 0·041 | 0·396 | 1·035 | 0·997 | 1·075 | 0·073 | 0·250 | 1·004 | 0·951 | 1·060 | 0·881 | 1·000 |
| ADHD | 1·133 | 1·133 | 1·088 | 9·98E-10 | **2·4E-08** | 1·119 | 1·078 | 1·162 | 3·96E-09 | **6·34E-08** | 0·992 | 0·940 | 1·047 | 0·767 | 1·000 |
| Alcohol dependence disorder | 1·036 | 1·036 | 0·994 | 0·091 | 0·548 | 1·050 | 1·010 | 1·091 | 0·015 | 0·083 | 0·993 | 0·940 | 1·049 | 0·801 | 1·000 |
| Anxiety disorders meta-analysis: factor scores | 1·044 | 1·044 | 1·002 | 0·038 | 0·396 | 1·000 | 0·963 | 1·039 | 0·982 | 1·000 | 0·953 | 0·903 | 1·006 | 0·079 | 0·597 |
| Anxiety disorders meta-analysis: case-control | 0·999 | 0·999 | 0·960 | 0·948 | 1·000 | 1·044 | 1·005 | 1·083 | 0·026 | 0·122 | 1·042 | 0·988 | 1·100 | 0·131 | 0·656 |
| Bipolar disorder | 1·036 | 1·036 | 0·995 | 0·086 | 0·548 | 1·104 | 1·063 | 1·147 | 3·20E-07 | **3·84E-06** | 1·057 | 1·001 | 1·116 | 0·045 | 0·546 |
| MDD | 1·170 | 1·170 | 1·123 | 3·33E-14 | **1·60E-12** | 1·218 | 1·173 | 1·266 | 2·20E-16 | **2·20E-16** | 1·048 | 0·992 | 1·106 | 0·094 | 0·597 |
| Schizophrenia | 1·131 | 1·131 | 1·084 | 8·64E-09 | **1·38E-07** | 1·143 | 1·098 | 1·188 | 3·27E-11 | **7·84E-10** | 1·009 | 0·955 | 1·066 | 0·756 | 1·000 |
|  |  |  |  |  |  |  |  |  |  |  |  |  |  |  |  |
| **Substance use phenotypes** |  |  |  |  |  |  |  |  |  |  |  |  |  |  |  |
| Lifetime cannabis use | 1·034 | 0·992 | 1·077 | 0·110 | 0·586 | 1·057 | 1·017 | 1·099 | 0·005 | **0·036** | 1·014 | 0·960 | 1·071 | 0·612 | 1·000 |
| Cigarettes per day | 0·976 | 0·938 | 1·016 | 0·229 | 0·845 | 0·991 | 0·954 | 1·029 | 0·643 | 1·000 | 1·010 | 0·956 | 1·066 | 0·732 | 1·000 |
| Daily alcohol use | 0·995 | 0·949 | 1·043 | 0·827 | 1·000 | 1·003 | 0·960 | 1·049 | 0·883 | 1·000 | 1·006 | 0·945 | 1·071 | 0·843 | 1·000 |
|  |  |  |  |  |  |  |  |  |  |  |  |  |  |  |  |
| **Cognitive trait** |  |  |  |  |  |  |  |  |  |  |  |  |  |  |  |
| Education attainment | 1·040 | 0·997 | 1·085 | 0·068 | 0·547 | 0·986 | 0·948 | 1·025 | 0·478 | 1·000 | 0·916 | 0·866 | 0·968 | 0·002 | 0·086 |
|  |  |  |  |  |  |  |  |  |  |  |  |  |  |  |  |
| **Personality traits** |  |  |  |  |  |  |  |  |  |  |  |  |  |  |  |
| Conscientiousness | 0·998 | 0·958 | 1·039 | 0·915 | 1·000 | 0·975 | 0·939 | 1·013 | 0·194 | 0·583 | 0·979 | 0·927 | 1·033 | 0·438 | 1·000 |
| Extraversion | 1·011 | 0·971 | 1·053 | 0·601 | 1·000 | 0·968 | 0·931 | 1·005 | 0·089 | 0·284 | 0·960 | 0·909 | 1·013 | 0·137 | 0·656 |
| Neuroticism IRT | 1·031 | 0·991 | 1·072 | 0·133 | 0·638 | 1·047 | 1·009 | 1·087 | 0·016 | 0·083 | 1·022 | 0·968 | 1·078 | 0·429 | 1·000 |
| Agreeableness | 0·979 | 0·940 | 1·018 | 0·287 | 0·983 | 0·992 | 0·955 | 1·029 | 0·656 | 1·000 | 0·998 | 0·946 | 1·053 | 0·950 | 1·000 |
| Aggression | 1·026 | 0·986 | 1·068 | 0·211 | 0·845 | 1·013 | 0·976 | 1·052 | 0·487 | 1·000 | 1·011 | 0·958 | 1·068 | 0·685 | 1·000 |
| Antisocial behaviour | 1·015 | 0·976 | 1·056 | 0·462 | 1·000 | 1·041 | 1·003 | 1·081 | 0·036 | 0·143 | 1·011 | 0·958 | 1·067 | 0·699 | 1·000 |
|  |  |  |  |  |  |  |  |  |  |  |  |  |  |  |  |
| **Physical traits** |  |  |  |  |  |  |  |  |  |  |  |  |  |  |  |
| Birth length | 1·026 | 0·984 | 1·069 | 0·227 | 0·845 | 0·958 | 0·923 | 0·995 | 0·028 | 0·122 | 0·939 | 0·889 | 0·992 | 0·024 | 0·391 |
| Birth weight | 1·020 | 0·979 | 1·062 | 0·344 | 1·000 | 1·038 | 1·000 | 1·078 | 0·052 | 0·192 | 1·019 | 0·965 | 1·076 | 0·498 | 1·000 |
| Adult height | 0·972 | 0·918 | 1·029 | 0·332 | 1·000 | 0·968 | 0·919 | 1·021 | 0·230 | 0·649 | 0·958 | 0·910 | 1·008 | 0·099 | 0·597 |
| Overweight | 0·995 | 0·956 | 1·036 | 0·823 | 1·000 | 1·017 | 0·980 | 1·057 | 0·368 | 0·982 | 1·019 | 0·965 | 1·076 | 0·498 | 1·000 |
| Extreme BMI | 1·006 | 0·967 | 1·047 | 0·761 | 1·000 | 1·056 | 1·018 | 1·097 | 0·004 | **0·036** | 1·052 | 0·996 | 1·110 | 0·067 | 0·597 |
| BMI | 0·983 | 0·943 | 1·023 | 0·398 | 1·000 | 1·052 | 1·013 | 1·093 | 0·009 | 0·062 | 1·075 | 1·018 | 1·135 | 0·009 | 0·228 |

*Note.* ^a,b^ These are the outputs from the same multinomial model, in which “Never self-harmed” was the reference group. ^c^ These outputs were derived from a different multinomial model, in which “NSSH” was the reference group. The *q*-values in bold are those that met the *q* < .05 threshold. OR = Odds Ratio.
