# Supplementary material for: Studying individual risk factors for self-harm in the UK Biobank: A polygenic scoring and Mendelian randomisation study
Source: PLoS Med. 2020 Jun 1;17(6):e1003137. doi: 10.1371/journal.pmed.1003137 (PMC7263593; doi:10.1371/journal.pmed.1003137)
Supplement: S6 Table — (DOCX) [file pmed.1003137.s012.docx]

**S6 Table. I^2^_GX_ statistics for the MR instruments in univariable MR analyses**

| Trait | I^2^_GX_ |
| --- | --- |
| ADHD | 94.9% |
| Alcohol dependence disorder | 93.0% |
| Bipolar disorder | 94.4% |
| Lifetime cannabis use | 94.7% |
| MDD | 94.7% |
| Schizophrenia | 95.7% |

Note. These I^2^_GX_ statistics are applicable to MR for both self-harm and SSH as outcomes.
