# Supplementary material for: Studying individual risk factors for self-harm in the UK Biobank: A polygenic scoring and Mendelian randomisation study
Source: PLoS Med. 2020 Jun 1;17(6):e1003137. doi: 10.1371/journal.pmed.1003137 (PMC7263593; doi:10.1371/journal.pmed.1003137)
Supplement: S7 Table — (DOCX) [file pmed.1003137.s013.docx]

| Exposure | N_snps_ | *ß* | 95% CI lower bound | 95% CI upper bound | p-value |
| --- | --- | --- | --- | --- | --- |
| ADHD | 496 | 0.001 | -0.001 | 0.003 | 0.180 |
| MDD | 496 | 0.010 | 0.006 | 0.014 | **5.48E-08** |
| Schizophrenia | 496 | 0.003 | 0.001 | 0.005 | **0.002** |

**S7 Table. Multivariable MR with self-harm as outcome, using a genome-wide *p*-value threshold (*p* < 5e-8) to select the instruments.**

Note. Since using a liberal *p*-value threshold might increase the risk for pleiotropy, we repeated the analysis using a more stringent *p*-value threshold (*p* < 5e-8) for schizophrenia. We did not use the same threshold for MDD and ADHD because applying such threshold only resulted in 2 and 13 MR instruments respectively.
