# Supplementary material for: Studying individual risk factors for self-harm in the UK Biobank: A polygenic scoring and Mendelian randomisation study
Source: PLoS Med. 2020 Jun 1;17(6):e1003137. doi: 10.1371/journal.pmed.1003137 (PMC7263593; doi:10.1371/journal.pmed.1003137)
Supplement: S8 Table — (DOCX) [file pmed.1003137.s014.docx]

**S8 Table. Univariable MR results with SSH as the outcome.**

| Exposure | Method | N_snps_ | ß | 95% CI lower bound | 95% CI upper bound | p-value |
| --- | --- | --- | --- | --- | --- | --- |
| ADHD | IVW | 244 | 0.014 | -0.006 | 0.034 | 0.168 |
|  | MR RAPS |  | 0.015 | -0.006 | 0.037 | 0.165 |
|  | Weighted median |  | 0.014 | -0.015 | 0.043 | 0.361 |
|  | MR–Egger regression |  | -0.002 | -0.066 | 0.062 | 0.949 |
|  | MR–Egger intercept |  | 0.001 | -0.004 | 0.007 | 0.602 |
| Alcohol dependence disorder | IVW | 86 | 0.009 | -0.009 | 0.027 | 0.325 |
|  | MR RAPS |  | 0.011 | -0.009 | 0.031 | 0.291 |
|  | Weighted median |  | 0.010 | -0.016 | 0.036 | 0.433 |
|  | MR–Egger regression |  | -0.016 | -0.054 | 0.022 | 0.414 |
|  | MR–Egger intercept |  | 0.005 | -0.002 | 0.011 | 0.146 |
| Bipolar disorder | IVW | 77 | -0.003 | -0.024 | 0.018 | 0.791 |
|  | MR RAPS |  | -0.004 | -0.028 | 0.020 | 0.757 |
|  | Weighted median |  | -0.003 | -0.033 | 0.027 | 0.860 |
|  | MR–Egger regression |  | -0.023 | -0.116 | 0.070 | 0.629 |
|  | MR–Egger intercept |  | 0.003 | -0.009 | 0.015 | 0.664 |
| Lifetime cannabis use | IVW | 85 | -0.003 | -0.021 | 0.015 | 0.737 |
|  | MR RAPS |  | -0.007 | -0.027 | 0.013 | 0.496 |
|  | Weighted median |  | 0.008 | -0.020 | 0.036 | 0.598 |
|  | MR–Egger regression |  | -0.018 | -0.058 | 0.022 | 0.378 |
|  | MR–Egger intercept |  | 0.003 | -0.004 | 0.009 | 0.412 |
| MDD | IVW | 239 | -0.007 | -0.036 | 0.022 | 0.655 |
|  | MR RAPS |  | -0.011 | -0.043 | 0.020 | 0.481 |
|  | Weighted median |  | 0.008 | -0.049 | 0.064 | 0.783 |
|  | MR–Egger regression |  | -0.024 | -0.084 | 0.036 | 0.427 |
|  | MR–Egger intercept |  | 0.001 | -0.003 | 0.005 | 0.509 |
| Schizophrenia | IVW | 1003 | 0.022 | 0.011 | 0.033 | **1.42E-04** |
|  | MR RAPS |  | 0.024 | 0.012 | 0.037 | **1.06E-04** |
|  | Weighted median |  | 0.043 | 0.026 | 0.061 | **1.39E-06** |
|  | MR–Egger regression |  | 0.061 | 0.029 | 0.092 | **1.68E-04** |
|  | MR–Egger intercept |  | -0.003 | -0.005 | -0.001 | **0.010** |
|  |  |  |  |  |  |  |
